# Supplementary material for: Utilization of modern contraceptive methods and its determinants among youth in Myanmar: Analysis of Myanmar Demographic and Health Survey (2015-2016)
Source: PLoS One. 2021 Oct 6;16(10):e0258142. doi: 10.1371/journal.pone.0258142 (PMC8494330; doi:10.1371/journal.pone.0258142)
Supplement: Attachment — Submitted filename: Response to Reviewers.docx [file pone.0258142.s002.docx]

**Responses to Reviewer 1**

Dear Reviewer

Thank you very much for pointing out some mistakes of us. I revised according to your comments.

1. Line 127-128 Why was “the number of modern contraceptive methods known” not categorized? I think it was because reviewers suggested revising the category of this variable in the first review. In Table 5 and Table 6 of the revised manuscript, the value of this variable did not describe any reference category. If it intended to use continuous variable, the result of logistic regression on this continuous variable should be completely described in Table 5 and Table 6. On the other hand, if it used binary variable, the variable should be categorized into at least 2 categories. For example, “0” for nothing known, and “1-11” for knowing one and more methods or “no”/ “yes” category.

**Author's response** – We received the comments from the Academic editor not to categorize the number of modern contraceptive methods known; hence, we revised accordingly and treated it as a continuous variable. To clear the reader, we added a sentence at **Line 128-129** "we treated this variable as a continuous variable."

Since we treated this variable as a continuous independent variable, we don't need a reference category for binary logistic regression analysis for the dependent variable (contraceptive utilization) in Table 5 and Table 6. We interpreted the results like how much odds of contraceptive utilization were increased or decreased as one more method knew.

2. In Line 338, it described “most knew 4.5 to 8.8 contraceptive methods”. Numerically, it is true to describe the real value. But logically, the method should be counted in compete number. 4.5 or 8.8 Methods looks a little unrealistic. One method cannot be divided into 0.5 or 0.8 method. This is just optional.

**Author's response** – We agree with your comments since the number of known contraceptive methods was a discrete numerical variable. However, this finding was not our study's finding. We used the finding of reference number [32] to discuss knowledge of modern contraceptive methods.

3. Line 373 described dose-response relationship between knowledge and utilization. However, the manuscript did not describe the statistical values of this relationship in the result session. May be I misunderstood Table 6. My suggestion is to use a figure or illustration to describe “this dose-response relationship” for more understanding by readers.

**Author's response** – We performed a chi-square test for trend to describe this dose-response relationship and the results were described using Fig 3. Please see Fig 3 and the description of this figure in **Lines 227-230.**

4. Line 392-393 argued that youth from rich households utilized more contraceptives than ones from poor household. It looks contradictory to the finding. In Line 287, it stated that “Female youth from rich households were 32% less likely to use modern contraception than poor households”. Please review it and kindly keep consistency of manuscript.

**Author's response** – Thank you so much for pointing out this gross mistake. We revised this finding appropriately. Please see **Line 399.**

**Other minor corrections by Author**

1. In line 184 of the unmarked version, we replaced the words "three fourth" with "three-fourths".
2. In lines 260-261 of the unmarked version, we replaced the sentence "Youth from the hilly zone were 70% fewer odds to use modern contraception than those from the delta zone" with "Youth from the hilly zone had 70% fewer odds of using modern contraception than those from the delta zone".
